# Real-world data on aortic endografts: Gore Together aortic registry protocol

**DOI:** 10.3389/fcvm.2026.1803528

**Published:** 2026-08-28

**Authors:** Dai Yamanouchi, Manar Khashram, Dennis Gable, Ross Milner, Kak Khee Yeung, Timothy A. Resch, Dittmar Böckler, Chris Delaney, Ali Azizzadeh, Kazuo Shimamura, Raffi Qasabian, Sukgu Han, Yuri Feito, Santi Trimarchi

**Affiliations:** 1 Division of Vascular Surgery, University of Wisconsin School of Medicine and Public Health, Madison, WI, United States; 2 Department of Vascular Surgery, Fujita Health University, Toyoake, Japan; 3 Department of Vascular and Endovascular Surgery, Waikato Hospital, University of Auckland Hamilton, Hamilton, New Zealand; 4 Department of Vascular and Endovascular Surgery, Baylor Scott and White Heart Hospital Plano, Plano, TX, United States; 5 The Division of Vascular Surgery and Endovascular Therapy, University of Chicago Pritzker School of Medicine, Chicago, IL, United States; 6 Department of Surgery, Amsterdam University Medical Centers, University of Amsterdam, Amsterdam, Netherlands; 7 Department of Vascular Surgery, Faculty of Health Sciences, Copenhagen University Hospital: Rigshospitalet, Denmark and Copenhagen University, Copenhagen, Denmark; 8 Department of Vascular and Endovascular Surgery, University Hospital Heidelberg, Heidelberg, Germany; 9 Department of Vascular and Endovascular Surgery, Flinders Medical Centre, Adelaide, SA, Australia; 10 College of Medicine and Public Health, Flinders University, Adelaide, SA, Australia; 11 Division of Vascular Surgery, Smidt Heart Institute and Randall Department of Surgery, Cedars-Sinai Medical Center, Los Angeles, CA, United States; 12 Department of Cardiovascular Surgery, The University of Osaka, Graduate School of Medicine Osaka, Japan; 13 Department of Cardiovascular Surgical Technology Innovation, The University of Osaka, Graduate School of Medicine, Osaka, Japan; 14 Royal Prince Alfred Hospital, Camperdown, Australia; 15 Division of Vascular Surgery and Endovascular Therapy, Keck Medical Center of University of Southern California, Los Angeles, CA, United States; 16 W. L. Gore & Associates, Flagstaff, AZ, United States; 17 Division of Vascular Surgery, Cardiac Thoracic Vascular Department, Fondazione IRCCS Ca’ Granda Ospedale Maggiore Policlinico, Milan, Italy; 18 Department of Clinical and Community Sciences, University of Milan, Milan, Italy

**Keywords:** clinical trial, EVAR, real world evidence (RWE), TEVAR, trial design

## Abstract

**Background:**

Post-market long-term surveillance data for aortic endovascular devices are essential due to evolving regulatory standards and clinical practice demands. Building upon the successful Global Registry for Endovascular Aortic Treatment (GREAT), comprehensive real-world data is required to address current gaps in long-term device performance and patient outcomes. Thus, the purpose of this manuscript is to describe the protocol, objectives, and anticipated outcomes of the Gore Together Aortic Registry, designed for extensive real-world evaluation of the performance and clinical outcomes of aortic endovascular devices.

**Methods:**

The Gore Together Aortic Registry is an international, prospective, observational registry initiated in response to increased regulatory emphasis on long-term post-market surveillance. This registry will enroll approximately 3,355 adult patients (≥18 years old) from over 170 global clinical centers, undergoing routine clinical care with GORE® aortic endovascular devices. Enrollment commenced based on standard clinical application, with data and imaging collection beginning at the time of hospitalization, and at each follow-up period.

**Ethics and dissemination:**

Primary registry outcomes include technical deployment success, measures of device or procedural adverse events, including endoleaks, as well as post-procedure reinterventions. Individual modules can tailor their reporting to address pertinent needs that may include dissection characterization, neurological complications or renal dysfunction. Follow-up is collected per standard of care with the potential to capture up to 10 years of data based upon device need. Patient enrollment is ongoing; no results are reported at this stage.

**Conclusion:**

The Gore Together Aortic Registry aims to deliver robust and granular real-world data regarding mid-term clinical outcomes of aortic endovascular devices, enhancing patient care strategies and meeting evolving global regulatory expectations. Results will guide clinical decisions, inform regulatory oversight, and potentially improve device safety and efficacy.

**Clinical trial registration:**

The individual modules of the Gore Together Aortic Registry are registered in ClinicalTrials.gov as:

1. https://clinicaltrials.gov/study/NCT06218875, identifier NCT06218875
2. https://clinicaltrials.gov/study/NCT06507865, identifier NCT06507865
3. https://clinicaltrials.gov/study/NCT06658730, identifier NCT06658730

## 1 Introduction

In 2010, a large observational registry named the Global Registry for Endovascular Aortic Treatment (GREAT; ClinicalTrials.gov Identifier: NCT01658787) was initiated to capture real-world clinical outcomes and severe adverse events associated with commercially available endovascular aortic devices. This registry enrolled data from over 5,000 patients across multiple international regions, including Europe, North America, South America, and Australia (1). The registry has provided substantial evidence, that have contributed to our understanding of endovascular treatment outcomes, such as device durability, secondary interventions, and patient survival (Supplement 1).

Since the establishment of the GREAT registry, global regulatory frameworks for medical devices have evolved, demanding rigorous post-market surveillance and comprehensive, real-world data collection to ensure long-term device safety and effectiveness (2–4). Notably, regulations such as the European Union's Medical Device Regulation (EU MDR 2017/745) and initiatives by the U.S. Food and Drug Administration (FDA), such as the National Evaluation System for Health Technology (NEST), emphasize continuous, detailed monitoring of clinical outcomes and patient safety in real-world settings (5). These regulatory shifts highlight the increasing demand for high-quality, real-world data to support clinical decision-making, post-market surveillance, and regulatory compliance.

In response to these regulatory demands and the need for improved patient-centered evaluations, a new international observational registry, the Gore Together Aortic Registry (Together Registry), was established. This registry is structured to systematically collect standardized clinical outcomes and patient-reported outcomes (PROs), including quality-of-life measures and symptom relief (6–8). By integrating standardized data collection methodologies and expanding geographical scope, the registry aims to enhance the understanding of clinical outcomes and long-term device performance, facilitating comparisons across patient populations and device types. In addition, imaging data will be included, allowing detailed interventional analysis, related outcomes, as well as providing future opportunities for robust morphological assessment of aneurysm behavior following endograft implantation.

Ethical considerations and adherence to stringent data privacy standards, such as the General Data Protection Regulation (GDPR) and the Health Insurance Portability and Accountability Act (HIPAA), are integral components of the Together Registry design. These rigorous standards ensure patient confidentiality and data integrity.

This manuscript provides a detailed overview of the Together Registry, describing its objectives, methodology, and anticipated contributions to clinical practice and regulatory frameworks through robust and comprehensive real-world data.

## 2 Methods and analysis

### 2.1 Study design

The Together Registry employs a prospective, observational, multicenter design, aiming to evaluate real-world outcomes of endovascular aortic interventions and foster collaboration across academic and community centers across the world. This registry systematically captures data across multiple device types utilized in the treatment of abdominal and thoracic aortic pathologies. It assesses the performance of multiple devices within Gore's Aortic portfolio across three distinct modules, including the GORE® EXCLUDER® Conformable AAA Endoprosthesis (EXCC), and GORE® EXCLUDER® Iliac Branch Endoprosthesis (IBE) [NCT06218875], GORE® TAG® Conformable Thoracic Stent Graft with ACTIVE CONTROL System (CTAG-AC) [NCT06658730] and GORE® TAG® Thoracic Branch Endoprosthesis (TBE) [NCT06507865]. Unlike randomized controlled trials, this registry does not prescribe specific procedural methodologies or interventions beyond routine clinical care, thus ensuring the collection of representative real-world clinical applications and outcomes from diverse practice settings.

### 2.2 Ethical considerations and regulatory compliance

The registry adheres to international research and regulatory guidelines, including Good Clinical Practice (GCP) as defined by ISO 14155:2020 and ICH E6 (R2), as well as regulatory requirements established by the FDA, the European Medicines Agency (EMA), the Ministry of Health, Labour and Welfare (MHLW) and other applicable health authorities. Institutional Review Board (IRB) or Ethics Committee (EC) approval is obtained for all participating sites, ensuring compliance with ethical principles and patient protection standards. Data governance is maintained through rigorous de-identification protocols and adherence to the General Data Protection Regulation (GDPR) and the Health Insurance Portability and Accountability Act (HIPAA), safeguarding patient confidentiality.

Protocol deviations will be systematically reviewed to confirm classification and ensure appropriate documentation. Any identified trends or concerns will undergo further evaluation and, when necessary, corrective measures or protocol modifications will be implemented to minimize recurrence, thereby maintaining consistency with regulatory and ethical standards. Adverse events and device deficiencies will be subject to systematic review by an Observational Safety Monitoring Board, which includes 3 to 5 experts in the field with extensive clinical experience. Reported events, including those from comparator groups, will be evaluated to identify potential safety signals, confirm coding accuracy, and determine whether further investigation or communication with stakeholders is required. Signals suggesting a potential health threat will prompt timely escalation to ensure patient safety and regulatory compliance. The registry protocol was approved by the Institutional Review Board or Independent Ethics Committee at each participating institution, and all participating sites complied with their respective local regulatory and ethical requirements.

### 2.3 Participants and enrollment

As a real-world registry and collaboration across academic and community centers, enrollment is structured to ensure comprehensive regional representation of diverse patient populations through a multi-site approach, encompassing 96 clinical centers for the EXCC/IBE module, 57centers for the CTAG-AC module, and 26 centers for the TBE module (Table 1). The total number of enrolled patients will vary by module. The EXCC/IBE module targets up to 2,000 patients (1,700 and 300, respectively), the CTAG-AC module aims to enroll 1,200 participants, and the TBE module includes approximately 155 subjects. Follow-up duration is tailored to the characteristics of the devices under investigation. The EXCC/IBE, and CTAG-AC modules follow patients for ten years post-procedure, whereas the TBE module has a follow-up period of two years. The duration of the latter was considered sufficient for assessing mid-term device performance, particularly in the context of evaluating regional variations in device utilization and procedural outcomes. This approach is supported by the existence of separate, ongoing longitudinal data collection efforts designed to capture long-term outcomes (9).

**Table 1:** Together aortic registry enrollment.

Devices	Clinical Sites	Enrollment Target			Total
	Total No. of Sites^a^	North America	EMEA^b^	Asia Pacific^c^	
GORE® EXCLUDER® EXCC module	96	790	505	405	1,700
GORE® EXCLUDER® IBE Module		110	45	145	300
GORE® TAG® Conformable with Active Control Thoracic Stent Graft Module	55	600	300	300	1,200
GORE® TAG® Thoracic Branch Endoprosthesis (TBE) Module	26	–	120	35	155

a This is an approximate number as the site selection process is ongoing.

b Europe, Middle East and Asia region.

c Australia, Japan, New Zealand.

### 2.4 Patient and public involvement

This research was conducted without patient or public involvement. Patients and members of the public were not involved in the design of the study, the selection of outcome measures, the conduct of the research, or the interpretation and dissemination of the results. The Together Registry is a prospective observational clinical registry designed to collect data arising from routine clinical care, and methodological decisions were driven by clinical and regulatory requirements. Participants provide informed consent for data collection in accordance with local ethics approvals.

### 2.5 Patient population and eligibility criteria

Patients eligible for inclusion in the registry are those requiring aortic intervention with a Gore aortic endovascular device as part of their standard clinical care. To ensure broad applicability and facilitate the collection of real-world data, inclusion criteria encompass individuals aged 18 years or older who have undergone or are planned to undergo treatment with an eligible device (Table 2). Subjects will be included irrespective of whether the device was used within or outside its labeled indications for use, thereby capturing the full spectrum of real-world practice patterns.

**Table 2:** Together registry inclusion and exclusion criteria.

Inclusion Criteria	Exclusion Criteria
1. Patient or legally authorized representative (LAR) provides written authorization and/or consent per institutional and geographical requirements. 2. Patient has been or is intended to be treated with an eligible registry device.^a^ 3. Patients ≥18 years of age at the time of informed consent signature.	1. Patient who is, at the time of consent, unlikely to be available for standard-of-care (SOC) follow-up visits, as defined by the site's guidelines and procedures. 2. Patients have exclusion criteria required by local law. 3. Patients are currently enrolled in or plans to enroll in any concurrent investigational drug and/or investigational device study within 12 months of Together Registry enrollment^b^. Subjects cannot be enrolled in another Together Registry module protocol.

a The intent to treat a patient with a Gore product must be made prior to soliciting for registry participation. If pre-procedure consent is not feasible due to emergent situation, consent from the subject or LAR prior to the time of discharge for the index procedure is acceptable.

b The term “study” does not apply to other observational registries or quality improvement projects. Collection of EXCC or IBE performance from interventional studies may be permissible provided device application is not investigational and there are no novel requirements that alter follow-up conduct (i.e., protocol-mandated interventions).

Written informed consent, following guidelines from the Declaration of Helsinki and Good Clinical Practice (GCP), is mandatory for participation, as it constitutes a fundamental ethical and regulatory requirement in clinical research. Prior to enrollment, all patients or their legally authorized representatives will receive comprehensive information regarding the study's objectives, procedures, potential risks and benefits, and their rights as research participants from a health care professional or trial designee at the respective clinical site. Participants retain the right to withdraw from the study at any stage time without impacting their medical care. Key exclusion criteria include anticipated inability to complete follow-up, concurrent enrollment in other investigational studies, and any legal or regulatory prohibitions affecting participation (Table 2). To widen the generalizability of the participants, acute patients could also be enrolled after implantation of an unplanned aortic devices in an emergency setting after providing written consented.

### 2.6 Intervention and data collection

Patients enrolled in the registry will undergo implantation of the designated Gore device through standard endovascular techniques, with procedural details recorded in an electronic case report form. The EXCC/IBE module focuses on the endovascular repair of abdominal and iliac aneurysms, with the EXCC device indicated for abdominal aortic aneurysms (AAA) and the IBE device designed for iliac aneurysm repair, including both unilateral and bilateral applications. The CTAG-AC module evaluates the performance of the device in the context of thoracic endovascular aortic repair (TEVAR), while the TBE module examines its use in zone 0∼2 coverage for aortic disease requiring preservation of supra-aortic trunk vessel perfusion. Procedural adjuncts, including additional stent grafts, cervical debranching, and embolization techniques, are employed at the discretion of the treating physician and recorded as part of the intervention data.

Data collection utilizes a validated electronic data capture platform (Medidata Rave®), ensuring consistency across participating sites. Imaging assessments, including computed tomography angiography (CTA), are encouraged, with clinical centers submitting pre- and post-operative scans for independent evaluation by Gore Imaging Sciences (GIS). GIS follows standardized, pre-defined measurement protocols for the evaluation of imaging endpoints, including endoleak classification and device migration, consistent with established core laboratory practices.

Clinical follow-up is structured to follow standard of care at each facility up to ten years for the EXCC/IBE and CTAG-AC modules. The TBE module follows a modified schedule, with assessments concluding at the two-year mark (Table 3).

**Table 3:** Participant timeline: schedule of enrollment and follow up assessment.^a^

Follow-Up Milestone	EXCC & IBE Module	CTAG-AC Module	TBE Module
Pre-procedure assessment	✓	✓	✓
Post-procedure (discharge)	✓	✓	✓
1 month	✓	✓	✓
6 months	✓	✓	-
1 year	✓	✓	✓
Annual follow-up (years 2−10)	✓	✓	-
Final follow-up	10 years	10 years	2 years

Follow-up is designed to assess device integrity, patient outcomes, and potential complications.

a Follow up visits are determined by treatment teams based on good clinical practice.

Independent oversight is provided by an Observational Safety Monitoring Board (OSMB), which assesses the continuing safety of current patients and those yet to be recruited and the ongoing scientific integrity of the registry. The OSMB will be at specified intervals throughout the study for each individual module. Device-related complications are classified according to standardized criteria and reported in accordance with global regulatory requirements.

### 2.7 Follow-up and clinical outcomes assessment

The registry evaluates a range of clinical and device-related outcomes, categorized as primary and secondary measures (Table 4). The primary outcomes of interest include technical deployment success, all-cause and lesion-related mortality, and device-related adverse events such as endoleaks, device migration, loss of branch patency, renal function deterioration, and neurological complications, including stroke, paraplegia, and paraparesis (Table 4). Secondary outcomes are device specific and encompass access-related complications, new-onset renal failure, false lumen remodeling in TEVAR-related registries, and health economic parameters, including hospital length of stay and ICU admission rates, depending on the module. Additionally, the EXCC/IBE module assesses quality of life measures, using the EQ-5D-5L, a validated instrument available in over 150 language versions through the EuroQol Research Foundation, ensuring applicability across the registry's geographically diverse participating centers (10, 11), incidence of erectile dysfunction, and buttock claudication as part of long-term follow-up.

**Table 4:** Primary and secondary outcomes for each module.

Primary Outcomes		
• Deployment Technical Success (index procedure only)^a^ Assessment of the following through all follow-up windows (unless indicated) ○ Mortality (all-cause, lesion-related) ○ Device and Treatment-related Adverse Events (AEs), including: ▪ Lesion rupture (treated area) ▪ Lesion enlargement (treated area) ▪ Endoleaks ▪ Device migration ▪ Loss of aortic/branch patency ▪ New onset renal failure (w/in 30 days of the procedure) ▪ Renal function deterioration ○ Serious Adverse Events (SAEs) ○ AEs with unknown causal relationships ○ Device integrity events (e.g., fracture, kinking, compression) ○ Reintervention		

Secondary Outcomes		
AA Module	TA Module	TB Module
Characterization of remote data collection to supplement follow-up activities (utilization, frequency by time, outcomes, contribution to total follow-up, need for elevation of care) New onset buttock claudication/erectile dysfunction	Access-related complications (index procedure only) Transient Ischemic Attack (TIA) New dissection With/without treatment False lumen status/perfusion/remodeling (dissection pathology)	Access-related complications (index endovascular procedure only) Transient Ischemic Attack (TIA) Life threatening bleed Upper extremity ischemia New dissection With/without treatment False lumen status/perfusion/remodeling (dissection pathology)

a Technical success is defined as successful access, delivery, and accurate deployment of the device to the intended location, and retrieval of the delivery system. The absence of any additional corrective procedure related to the device, procedure, or withdrawal of the delivery system. Events will not include interventions at the access site(s), lumbar drains to address spinal cord ischemia, or additional interventions to address non-treatment areas.

### 2.8 Minimum enrollment targets

Minimum enrollment targets for the EXCC/IBE modules were established based on an assumed outcome rate 10%, which is on the scale of expected endoleak (12). A small subset of 20% of all subjects (*n* = 600) represents both possible ad-hoc sub-analysis and a conservative estimate of subjects available at 10 years. With the full anticipated enrollment of 2,000 EXCC subjects, there is 80% power to detect a difference between 8.5% vs. 10% rates. For events with rarity of 1/350, there is greater than 80% probability to observe one or more among a small subset of 600 subjects and six or more events among the full 3,000 subjects. Among the full sample of 3,000 EXCC subjects, and an outcome with rate of 10%, a 95% confidence interval length would be 2.2% and among the reduced population of 600, the 95% confidence interval length would be 5.0%.

For the TA module, enrollment targets were derived assuming an outcome rate of 10%, which is on the scale of expected endoleak for this patient population (13). With respect to the anticipated overall registry of *N* = 1,200 subjects, a small subset of *n* = 120 (10%) represents both a possible *ad hoc* subgroup analysis sample as well as a conservative estimate of subjects available at 10 years. With an enrollment of *N* = 1,200, there is greater than 80% probability to observe one or more of an extremely rare events (1/700) and four or more of a rare event (1/200). This enrollment target supports population proportion estimation with exact confidence interval width of, for example, 3.5% for *P* = 10%, or a maximum of 5.7% for *P* = 50%. For subgroup analyses composed of 10% of the registry (*N* = 120), the exact confidence interval widths would be 11.5% for *P* = 10% and 18.5% for *P* = 50%.

Lastly, for the TB module, the minimum enrollment determination was based on detecting rare events and reasonable confidence interval widths for binomial proportion estimates applied to either the overall population or to subgroups. With an enrollment of *N* = 155, there is greater than 80% probability to observe one or more unusual (1/90) and four or more uncommon events (1/25). This enrollment target supports population proportion estimation with exact confidence interval width of, for example, 6.9% for *P* = 4%, or a maximum of 16.3% for *P* = 50%.

### 2.9 Statistical analysis

As a real-world registry, no statistical hypotheses are specified. By default, statistical testing will test for two-sided differences across arbitrary subgroups to better understand clinical outcomes. All data will be included in their appropriate analysis as intention to treat.

Statistical analyses will employ a combination of descriptive and inferential methodologies to ensure comprehensive evaluation of patient outcomes. Kaplan–Meier survival analyses estimate freedom from mortality, reintervention, and major adverse events over time. Cox proportional hazards models will be used to identify predictors of device failure and clinical complications, while multivariate regression models assess the influence of patient demographics, procedural variables, and anatomical characteristics on long-term outcomes. To provide contextual comparisons, the registry integrates historical data from prior observational studies, allowing for benchmarking of device performance against established clinical standards.

The contrast-enhanced computed tomography (CT) component of this study provides a robust foundation for quantitative imaging analysis and potential future applications of machine learning. Contrast CT offers high spatial and temporal resolution that enables precise assessment of vascular anatomy, device positioning, and procedural outcomes. Beyond its immediate diagnostic and procedural value, the imaging dataset has been structured to support the extraction of radiomic and morphological features that could inform predictive modeling. This framework allows for future implementation of advanced analytical approaches, including machine learning-based image analysis, to identify subtle imaging biomarkers and optimize outcome prediction in endovascular interventions.

### 2.10 Evidence dissemination plan

The Gore Together Aortic Registry has been designed with a comprehensive dissemination strategy to ensure timely communication of clinically meaningful findings to the scientific community, regulatory agencies, and healthcare stakeholders. Dissemination activities will occur through multiple channels, with oversight from a sponsored designated Publication steering committee to ensure scientific integrity and alignment with registry objectives.

The global clinical data dissemination plan will follow established principles outlined in the International Committee of Medical Journal Editors (ICMJE) recommendations (14) and Good Publication Practice guidelines (15) to ensure transparency, accuracy, and integrity in scientific communication. The plan encompasses a comprehensive strategy for the structured release of study outcomes to the scientific and clinical communities. Dissemination activities will include presentations at major international and regional congresses, submission of abstracts reporting interim and final analyses, and preparation of peer-reviewed manuscripts describing key procedural and clinical endpoints.

Manuscripts will be developed according to predefined milestones, including outcomes assessed at the index procedure, 30 days, 6 months, and 1 year, with continued analyses and publications planned throughout the 10-year follow-up period. Longitudinal reporting will enable continuous evaluation of device performance, durability, and long-term patient outcomes. All dissemination activities will be coordinated and approved by the study steering committee in collaboration with regional investigators to ensure consistency, scientific rigor, and adherence to authorship, data sharing, and publication standards. This structured dissemination framework is intended to promote transparency, support evidence-based clinical practice, and facilitate sustained knowledge exchange within the global endovascular community.

## 3 Discussion

Over the years, vascular registries have provided essential benchmarking data that have driven improvements in healthcare quality and patient outcomes (16, 17). Together builds upon insights from the earlier GREAT registry, which advanced understanding of clinical outcomes associated with endovascular aortic repair (12, 13, 18–28), and provided valuable evidence on the long-term effectiveness of endovascular repair techniques.

In response to the evolving regulatory landscape—particularly enhanced requirements from the European Union Medical Device Regulation (EU MDR) and U.S. Food and Drug Administration (FDA) initiatives such as the National Evaluation System for health Technology (NEST)—the Together Registry was designed to generate structured, high-quality real-world evidence. Its flexible yet standardized methodology enables comprehensive capture of clinical and patient-reported outcomes, aligning with contemporary best practices in medical device evaluation.

A defining feature of the Together Registry is the systematic inclusion of imaging data obtained across multiple international centers. Imaging enhances data robustness by enabling objective assessment of anatomical changes, device performance, and procedural outcomes over time. Standardized acquisition and analysis protocols facilitate reproducibility and future advanced analytics, including radiomic and machine learning approaches. By correlating imaging findings with clinical and patient-reported outcomes, the registry supports refinement of treatment algorithms, optimization of patient selection, and identification of imaging-based predictors of success or failure. This integration of imaging may ultimately inform evidence-based updates to clinical practice guidelines and shape future standards of care in endovascular therapy.

The registry's design ensures that generated data are directly relevant to clinical practice, supporting improved decision-making, safety monitoring, and personalized patient management. The inclusion of patient-reported outcomes strengthens the patient-centered focus by capturing quality of life, functional recovery, and symptom relief as key indicators of treatment success. Moreover, the registry's global scope promotes international data comparability and generalization of findings across diverse healthcare systems and patient populations. Together, these elements establish a comprehensive framework for developing globally applicable, evidence-based recommendations that advance the standard of care in endovascular intervention.

### 3.1 Limitations

Despite its strengths, the Together Registry has several inherent limitations. Its observational design may introduce selection bias arising from variability in clinical decision-making and patient selection across participating centers. The absence of randomized controls limits the ability to establish causal relationships between device use and clinical outcomes. Inconsistent follow-up adherence across sites may also affect the completeness and reliability of long-term data.

Although rigorous data collection methods are employed, the influence of unmeasured or unknown confounders cannot be fully excluded. Inflammatory biomarkers associated with post-implantation syndrome, including peak temperature, leukocyte count, and C-reactive protein, are not mandated data elements within the eCRF. As a result, the registry's capacity to systematically evaluate the contribution of post-implantation inflammatory responses to secondary outcomes such as hospital length of stay and ICU admission rate is limited. Dedicated prospective studies with standardized inflammatory marker capture would be required to fully characterize this relationship. While patient-reported outcomes (PROs) provide valuable insights into subjective treatment effects, variability in patient compliance and response interpretation may introduce additional bias. Differences in healthcare delivery systems, clinical practices, and patient populations across countries may further limit the generalizability of findings.

Additionally, data completeness and accuracy depend on timely and consistent reporting by participating centers. Despite centralized validation procedures and routine audits designed to ensure data integrity, variations in documentation practices and resource availability may still affect overall data quality.

Finally, the 10-year follow-up period, while necessary to characterize long-term device performance, is inherently susceptible to patient attrition. Although a dedicated clinical operations team is engaged with participating sites to support retention, loss to follow-up remains a potential source of bias that may limit the completeness of long-term outcome data.

## 4 Conclusion

The Together Registry is strategically positioned to address critical clinical and regulatory needs through robust real-world evidence generation. By integrating insights from previous registries, implementing standardized data collection practices, including imaging, and incorporating patient-reported outcomes, this registry will enhance the understanding of long-term device performance and patient outcomes. Ultimately, it is expected to inform clinical practice, support regulatory compliance, and improve patient-centered care in the treatment of aortic disease.

## Protocol & statistical analysis plan

The study protocol and statistical analysis plan will be made available upon reasonable request to the study sponsor, consistent with institutional policies and protection of proprietary information. These documents will not be posted publicly, but qualified researchers or regulatory bodies may request access for purposes such as verification of methods or regulatory review. Any sharing will occur only after appropriate confidentiality safeguards are in place.

## Data sharing

Patients and the public were not involved in the design, conduct, reporting, or dissemination plans of this research. Deidentified individual participant data related to the primary and secondary endpoints will be made available to investigators with a methodologically sound research proposal. Data will be shared upon reasonable request and subject to a data sharing agreement.

## Dissemination policy

The trial results will be communicated through multiple coordinated channels to ensure transparency and broad accessibility. Summary results will be posted in the appropriate clinical trial registry within required timelines, and the primary findings will be submitted for publication in a peer reviewed journal following established reporting standards. Results will also be presented at relevant scientific and professional meetings to support timely exchange within the medical community. Plain language summaries will be prepared for trial participants and the public to promote clear understanding of the study outcomes, consistent with ethical recommendations supporting accessible communication. Additional dissemination to healthcare professionals and stakeholders will occur through scientific presentations and institutional communications, with all public-facing materials reflecting accurate and non-misleading interpretation of the evidence.

## References

[1] Loa J Dubenec S Cao P Milner R Silveira PG Trimarchi S. The gore global registry for endovascular aortic treatment: objectives and design. Ann Vasc Surg. (2016) 31:70–6. 10.1016/j.avsg.2015.08.024 26616496

[2] Raj N Fernandes S Charyulu NR Dubey A SR G Hebbar S. Postmarket surveillance: a review on key aspects and measures on the effective functioning in the context of the United Kingdom and Canada. Ther Adv Drug Saf. (2019) 10:2042098619865413. 10.1177/2042098619865413 31384423 PMC6661791

[3] Luff MK Sedrakyan A Ansari SA Siddiqui AH Liebeskind DS. Postmarket surveillance of neuroendovascular devices. Stroke: Vasc Interv Neurol. (2024) 4(2):e001081. 10.1161/SVIN.123.001081 41583580 PMC12778473

[4] Fraser AG. Postmarket surveillance of high-risk medical devices needs transparent, comprehensive and independent registries. BMJ Surg Interv Health Technol. (2020) 2(1):e000065. 10.1136/bmjsit-2020-000065 35051246 PMC8647614

[5] U.S. Department of Health and Human Services. Food and Drug Administration. Use of Real-World Evidence to Support Regulatory Decision-Making for Medical Devices: Guidance for Industry and Food and Drug Administration Staff. Washington, DC: Office of Surveillance and Biometrics (2017). p. 1–24.

[6] Matts ST Webber CM Bocell FD Caldwell B Chen AL Tarver ME. Inclusion of patient-reported outcome instruments in US FDA medical device marketing authorizations. J Patient Rep Outcomes. (2022) 6(1):38. 10.1186/s41687-022-00444-z 35441987 PMC9021347

[7] Zannad F Alikhaani J Alikhaani S Butler J Gordon J Jensen K. Patient-reported outcome measures and patient engagement in heart failure clinical trials: multi-stakeholder perspectives. Eur J Heart Fail. (2023) 25(4):478–87. 10.1002/ejhf.2828 36924142

[8] Zhang B Shankara SB Guo J Zhang H. Pivotal clinical trials with patient-reported outcome measures in premarket approval applications for high-risk medical devices from 2005 to 2018: review, examples, and regulatory considerations. Contemp Clin Trials. (2022) 116:106757. 10.1016/j.cct.2022.106757 35398250

[9] U.S. Food and Drug Administration. Post-Approval Studies (PAS) Database. Washington, D.C.: U.S. Department of Health and Human Services (2025). Available online at: https://www.accessdata.fda.gov/scripts/cdrh/cfdocs/cfPMA/pma_pas.cfm (Accessed January 7, 2026).

[10] Herdman M Gudex C Lloyd A Janssen MF Kind P Parkin D. Development and preliminary testing of the new five-level version of EQ-5D (EQ-5D-5L). Qual Life Res. (2011) 20(10):1727–36. 10.1007/s11136-011-9903-x 21479777 PMC3220807

[11] Mv R Janssen B Stolk E Boye KS Herdman M Kennedy-Martin M. EuroQol Research Foundation. EQ-5D-5L User Guide. The Netherlands: EuroQol Research Foundation (2025).

[12] Long C Katsargyris A Milner R Verhoeven E Investigators G. Five-Year results for abdominal aortic aneurysm repair with the GORE(R) EXCLUDER(R) device: insights from the gore global registry for endovascular aortic treatment (GREAT). Ann Vasc Surg. (2024) 106:247–54. 10.1016/j.avsg.2024.03.016 38815908

[13] Gable DR Verhoeven E Trimarchi S Böckler D Milner R Dubenec S. Endovascular treatment for thoracic aortic disease from the global registry for endovascular aortic treatment. J Vasc Surg. (2024) 79(5):1044–56 e1. 10.1016/j.jvs.2023.12.040 38154605

[14] International Committee of Medical Journal Editors. Recommendations for the Conduct, Reporting, Editing Publication of Scholarly Work in Medical Journals. Philadelphia, PA: American College of Physicians (2025). 10.12771/emj.2024.e48 PMC12093628 40704003

[15] DeTora LM Toroser D Sykes A Vanderlinden C Plunkett FJ Lane T. Good publication practice (GPP) guidelines for company-sponsored biomedical research: 2022 update. Ann Intern Med. (2022) 175(9):1298–304. 10.7326/M22-1460 36037471

[16] Cronenwett JL. Registries, research, and quality improvement. Eur J Vasc Endovasc Surg. (2020) 59(4):503–9. 10.1016/j.ejvs.2020.02.014 32179003

[17] Trimarchi S. The society for vascular surgery thoracic endovascular aortic repair guidelines support thoracic endovascular aortic repair as the primary therapy for the treatment of thoracic aortic aneurysms. J Vasc Surg. (2021) 73(1S):53S–4. 10.1016/j.jvs.2020.07.070 33349349

[18] Atkins E Milner R Delaney CL. Global registry for endovascular aortic treatment P. Raised BMI is associated with fewer type I endoleaks in patients treated with the gore excluder device: data from the global registry for endovascular aortic treatment (GREAT). J Cardiovasc Surg. (2023) 64(5):513–20. 10.23736/S0021-9509.23.12572-9 37458731

[19] Boutrous ML Peterson BG Smeds MR. Predictors of aneurysm sac shrinkage utilizing a global registry. Ann Vasc Surg. (2021) 71:40–7. 10.1016/j.avsg.2020.08.110 32889165

[20] Alvarez Marcos F Coto L Camblor Santervas JM A L Zanabili Al-Sibbai AA Alonso Perez M. Five year post-endovascular aneurysm repair aneurysm sac evolution in the GREAT registry: an insight in diabetics using propensity matched controls. Eur J Vasc Endovasc Surg. (2024) 67(6):912–22. 10.1016/j.ejvs.2023.10.033 37898359

[21] Schneider DB Milner R Heyligers JMM Chakfe N Matsumura J. Outcomes of the GORE iliac branch endoprosthesis in clinical trial and real-world registry settings. J Vasc Surg. (2019) 69(2):367–77 e1. 10.1016/j.jvs.2018.05.200 30064841

[22] Giese A Heyligers JMM Milner R. Five-year outcomes for bell bottom, iliac branch endoprosthesis, and coil and cover approaches from the GREAT registry. J Vasc Surg. (2024) 79(6):1369–78. 10.1016/j.jvs.2024.01.212 38316346

[23] Howard DPJ Marron CD Sideso E Puckridge PJ Verhoeven ELG Spark JI. Editor’s choice - influence of proximal aortic neck diameter on durability of aneurysm sealing and overall survival in patients undergoing endovascular aneurysm repair. Real world data from the gore global registry for endovascular aortic treatment (GREAT). Eur J Vasc Endovasc Surg. (2018) 56(2):189–99. 10.1016/j.ejvs.2018.03.027 29764709

[24] Piffaretti G Grassi V Lomazzi C Weaver F Mandigers T Bissacco D. Endovascular treatment of infected aortic aneurysms: a retrospective multicenter analysis from the GORE®GREAT registry study. Int J Endovasc Treat Innov Tech. (2024) 5(2):49–56. 10.61797/ijetit.v5i2.347

[25] Payne D Böckler D Weaver F Milner R Magee GA Azizzadeh A. Five-year outcomes of endovascular treatment for aortic dissection from the global registry for endovascular aortic treatment. J Vasc Surg. (2024) 80(4):1035–44. 10.1016/j.jvs.2024.05.055 38825212

[26] Barry IP Barns M Verhoeven E Wong J Dubenec S Heyligers JM. Excluder stent graft-related outcomes in patients with aortic neck anatomy outside of instructions for use (IFU): within the Global Registry for Endovascular Aortic Treatment (GREAT): Mid-term Follow-Up Results. Ann Vasc Surg. (2021) 76:222–31. 10.1016/j.avsg.2021.04.032 34182115

[27] Bissacco D Domanin M Weaver FA Azizzadeh A Miller CC Gable DR. Differences in mid-term outcomes between patients undergoing thoracic endovascular aortic repair for aneurysm or acute aortic syndromes: report from the global registry for endovascular aortic treatment. J Endovasc Ther. (2022) 29(5):731–8. 10.1177/15266028211064819 34911391

[28] Charlton-Ouw KM Ikeno Y Bokamper M Zakhary E Smeds MR, participants G. Aortic endograft sizing and endoleak, reintervention, and mortality following endovascular aneurysm repair. J Vasc Surg. (2021) 74(5):1519–26 e2. 10.1016/j.jvs.2021.04.045 33940075

