# Supplementary material for: Real-world data on aortic endografts: Gore Together aortic registry protocol
Source: Front Cardiovasc Med. 2026 Aug 28;13:1803528. doi: 10.3389/fcvm.2026.1803528 (PMC13561764; doi:10.3389/fcvm.2026.1803528)
Supplement: Supplementary file 1 [file Supplementaryfile1.docx]

Supplementary Material

# 1 Supplementary Table 1: Current list of sites involved in the Gore Together Aortic Registry.*

|  | TGR  23-02AA | TGR  23-02TA | TGR  23-02TB | Grand Total |
| --- | --- | --- | --- | --- |
| Australia | **6** |  | **4** | **10** |
| Bayside Health | 1 |  | 1 | 2 |
| Flinders Medical Centre Vascular Unit | 1 |  | 1 | 2 |
| Gold Coast Hospital and Health Service | 1 |  |  | 1 |
| John Hunter Hospital | 1 |  |  | 1 |
| Royal North Shore Hospital |  |  | 1 | 1 |
| Royal Perth Hospital | 1 |  | 1 | 2 |
| Royal Prince Alfred Hospital | 1 |  |  | 1 |
| Austria | **1** |  | **1** | **2** |
| Medizinische Universitat Wien | 1 |  | 1 | 2 |
| Denmark |  | **1** | **1** | **2** |
| Rigshospitalet |  | 1 | 1 | 2 |
| Finland | **2** |  |  | **2** |
| Helsinki University Hospital (HYKS III) | 1 |  |  | 1 |
| Tampereen Yliopistollinen Sairaala / Tampere University Hospital | 1 |  |  | 1 |
| France |  | **1** |  | **1** |
| Centre Hospitalier Universitaire Strasbourg |  | 1 |  | 1 |
| Germany | **3** | **3** | **4** | **10** |
| St. Franziskus Hospital | 1 |  |  | 1 |
| Universitätsklinikum Schleswig-Holstein |  |  | 1 | 1 |
| Universitatsklinikum Ulm |  | 1 |  | 1 |
| Universitatsmedizin Frankfurt |  | 1 |  | 1 |
| Universitatsmedizin Gottingen | 1 |  |  | 1 |
| University Hospital Munster |  |  | 1 | 1 |
| University Medical Center Hamburg-Eppendorf (UKE) |  |  | 1 | 1 |
| University of Heidelberg |  |  | 1 | 1 |
| University of Regensburg - Regensburg University Hospital |  | 1 |  | 1 |
| Vivantes Netzwerk für Gesundheit GmbH | 1 |  |  | 1 |
| Greece |  | **1** | **2** | **3** |
| Evangelismos General Hospital |  |  | 1 | 1 |
| Laiko General Hospital of Athens |  | 1 | 1 | 2 |
| Israel | **1** |  |  | **1** |
| Tel Aviv Sourasky Medical Center | 1 |  |  | 1 |
| Italy | **5** | **2** | **5** | **12** |
| AORN S.Anna e S. Sebastiano |  | 1 |  | 1 |
| ASST Spedali Civili di Brescia |  |  | 1 | 1 |
| Azienda Ospedaliera "G. Brotzu" |  |  | 1 | 1 |
| Azienda Ospedaliera Policlinico Sant'Orsola Malpighi |  |  | 1 | 1 |
| Azienda Ospedaliera Universitaria Integrata Verona | 1 |  |  | 1 |
| Azienda Ospedaliero Universitaria delle Marche | 1 |  |  | 1 |
| Azienda Ospedaliero Universitaria Policlinico di Bari | 1 |  |  | 1 |
| Azienda Ospedaliero-Universitaria di Padova |  |  | 1 | 1 |
| Fondazione IRCCS Policlinico San Matteo | 1 |  |  | 1 |
| IRCCS Policlinico di Milano |  | 1 |  | 1 |
| Ospedale San Giovanni Molinette | 1 |  |  | 1 |
| Ospedale San Martino |  |  | 1 | 1 |
| Japan | **19** | **10** |  | **29** |
| Aichi Medical University Hospital | 1 |  |  | 1 |
| Asahikawa Medical University Hospital | 1 |  |  | 1 |
| Fujita Health University Hospital | 1 | 1 |  | 2 |
| Hamamatsu University Hospital | 1 | 1 |  | 2 |
| Hiroshima City Hiroshima Citizens Hospital |  | 1 |  | 1 |
| Jichi Medical University Hospital | 1 |  |  | 1 |
| Kansai Medical University Hospital | 1 |  |  | 1 |
| Keio University Hospital | 1 |  |  | 1 |
| Kindai University Hospital |  | 1 |  | 1 |
| Kobe University Hospital | 1 |  |  | 1 |
| Kyushu University Hospital | 1 |  |  | 1 |
| Matsuyama Red Cross Hospital | 1 |  |  | 1 |
| Nagoya University Hospital | 1 | 1 |  | 2 |
| Nara Medical University Hospital | 1 | 1 |  | 2 |
| Oita University Hospital | 1 |  |  | 1 |
| Shizuoka City Shizuoka Hospital | 1 | 1 |  | 2 |
| The Jikei University Hospital | 1 | 1 |  | 2 |
| The Sakakibara Heart Institute of Okayama | 1 |  |  | 1 |
| The University of Osaka Hospital | 1 | 1 |  | 2 |
| The University of Tokyo Hospital | 1 |  |  | 1 |
| University Hospital, Kyoto Prefectural University of Medicine | 1 |  |  | 1 |
| University of Miyazaki Hospital |  | 1 |  | 1 |
| Netherlands | **4** |  | **2** | **6** |
| Amsterdam UMC Research BV | 1 |  | 1 | 2 |
| Radboud UMC |  |  | 1 | 1 |
| Stichting Elisabeth-TweeSteden Ziekenhuis | 1 |  |  | 1 |
| VieCuri Medical Center | 1 |  |  | 1 |
| Zuyderland Medical Center | 1 |  |  | 1 |
| New Zealand | **2** |  | **1** | **3** |
| Auckland District Health Board Charitable Trust | 1 |  | 1 | 2 |
| Clinical Trials New Zealand Ltd. | 1 |  |  | 1 |
| Saudi Arabia |  | **1** |  | **1** |
| King Faisal Specialist Hospital and Research Center |  | 1 |  | 1 |
| Spain | **3** | **1** | **1** | **5** |
| Hospital De La Santa Creu i Sant Pau | 1 |  |  | 1 |
| Hospital Universitari de Bellvitge |  | 1 |  | 1 |
| Hospital Universitario de Gran Canaria Dr Negrín | 1 |  |  | 1 |
| Hospital Universitario Miguel Servet |  |  | 1 | 1 |
| Hospital Universitario Virgen del Rocio | 1 |  |  | 1 |
| Sweden | **1** | **1** | **1** | **3** |
| Karolinska University Hospital | 1 |  |  | 1 |
| Skanes Universitetssjukhus - Malmo |  |  | 1 | 1 |
| Uppsala University |  | 1 |  | 1 |
| United Kingdom | **4** | **1** | **2** | **7** |
| Bristol NHS Foundation Trust |  | 1 |  | 1 |
| Countess of Chester Hospitals NHS Foundation Trust | 1 |  |  | 1 |
| Glenfield Leicester Hospital | 1 |  |  | 1 |
| Guy's and St Thomas', NHS Foundation Trust | 1 |  | 1 | 2 |
| St. Mary's Hospital, Imperial College Healthcare, NHS Trust | 1 |  | 1 | 2 |
| United States of America | **41** | **24** |  | **65** |
| Atlanta VA Medical Center | 1 |  |  | 1 |
| Augusta University | 1 | 1 |  | 2 |
| Baylor Research Institute | 1 | 1 |  | 2 |
| Beth Israel Deaconess Medical Center, Inc. | 1 | 1 |  | 2 |
| CAMC Health Education and Research Institute, Inc. | 1 |  |  | 1 |
| Cedars-Sinai Medical Center |  | 1 |  | 1 |
| Columbia Medical Center of Plano | 1 | 1 |  | 2 |
| Corewell Health System | 1 |  |  | 1 |
| CoxHealth | 1 |  |  | 1 |
| CVT Surgical Center | 1 |  |  | 1 |
| Duke University Medical Center | 1 | 1 |  | 2 |
| Emory University | 1 |  |  | 1 |
| Endeavor Health | 1 |  |  | 1 |
| Forsyth Medical Center | 1 |  |  | 1 |
| Hackensack UMC |  | 1 |  | 1 |
| Indiana University |  | 1 |  | 1 |
| Inova Health Care Services | 1 |  |  | 1 |
| Intermountain Medical Center | 1 | 1 |  | 2 |
| Leland Stanford Junior University | 1 | 1 |  | 2 |
| Mayo Clinic - Rochester | 1 |  |  | 1 |
| McLaren Health Care Corporation | 1 |  |  | 1 |
| Medical College of Wisconsin, Inc. | 1 |  |  | 1 |
| MedStar Health Research Institute |  | 1 |  | 1 |
| MH Mission Hospital | 1 |  |  | 1 |
| Minneapolis Heart Institute Foundation - Abbott Northwestern Hospital | 1 |  |  | 1 |
| Mount Sinai Medical Center of Florida | 1 |  |  | 1 |
| Portland Health Care System VA | 1 |  |  | 1 |
| Prisma Health - Upstate | 1 |  |  | 1 |
| Research Foundation SUNY Buffalo | 1 |  |  | 1 |
| Sentara Medical Group | 1 | 1 |  | 2 |
| St. Vincent Medical Group, Inc. | 1 |  |  | 1 |
| The Curators of the University of Missouri | 1 |  |  | 1 |
| The Hitchcock Foundation | 1 | 1 |  | 2 |
| The University of Texas Southwestern Medical Center at Dallas | 1 |  |  | 1 |
| Thomas Jefferson University |  | 1 |  | 1 |
| University Hospitals - Cleveland Medical Center | 1 | 1 |  | 2 |
| University of Arizona |  | 1 |  | 1 |
| University of California - Irvine | 1 |  |  | 1 |
| University of Chicago Hospital | 1 |  |  | 1 |
| University of Florida - Gainesville |  | 1 |  | 1 |
| University of Louisville | 1 |  |  | 1 |
| University of Maryland - Baltimore | 1 | 1 |  | 2 |
| University of North Carolina at Chapel Hill | 1 | 1 |  | 2 |
| University of Pennsylvania | 1 | 1 |  | 2 |
| University of South Florida | 1 | 1 |  | 2 |
| University of Southern California | 1 | 1 |  | 2 |
| University of Utah | 1 |  |  | 1 |
| University of Washington |  | 1 |  | 1 |
| University of Wisconsin System |  | 1 |  | 1 |
| Wake Forest University Health Sciences | 1 |  |  | 1 |
| Grand Total | **92** | **46** | **24** | **162** |

***Displays Active sites as of 27-Jul-26**

# 2 Supplementary Table 2: List of Principal Investigations involved in the Gore Together Aortic Registry.*

|  | TGR  23-02AA | TGR  23-02TA | TGR  23-02TB | Grand Total |
| --- | --- | --- | --- | --- |
| Australia | **6** |  | **4** | **10** |
| Chris Delaney | 1 |  | 1 | 2 |
| Jacky Loa | 1 |  |  | 1 |
| Nishath Altaf | 1 |  | 1 | 2 |
| Thodur Vasudevan | 1 |  | 1 | 2 |
| Venu Bhamidi | 1 |  |  | 1 |
| Vikram Puttaswamy |  |  | 1 | 1 |
| Zoheb Williams | 1 |  |  | 1 |
| Austria | **1** |  | **1** | **2** |
| Florian Wolf | 1 |  | 1 | 2 |
| Denmark |  | **1** | **1** | **2** |
| Timothy Resch |  | 1 | 1 | 2 |
| Finland | **2** |  |  | **2** |
| Pekka-Sakari Aho | 1 |  |  | 1 |
| Velipekka Suominen | 1 |  |  | 1 |
| France |  | **1** |  | **1** |
| Salome Kuntz |  | 1 |  | 1 |
| Germany | **3** | **3** | **4** | **10** |
| Alexander Oberhuber |  |  | 1 | 1 |
| Bernd Muehling |  | 1 |  | 1 |
| Dittmar Boeckler |  |  | 1 | 1 |
| Eberhard Grambow | 1 |  |  | 1 |
| Fiona Rohlffs |  | 1 |  | 1 |
| Guenay Kalender | 1 |  |  | 1 |
| Kyriakos Oikonomou |  | 1 |  | 1 |
| Martin Austermann | 1 |  |  | 1 |
| Philipp Schaefer |  |  | 1 | 1 |
| Tilo Koelbel |  |  | 1 | 1 |
| Greece |  | **1** | **2** | **3** |
| Chris Klonaris |  | 1 | 1 | 2 |
| Theodoros Kratimenos |  |  | 1 | 1 |
| Israel | **1** |  |  | **1** |
| Basheer Sheikh-Yousif | 1 |  |  | 1 |
| Italy | **5** | **2** | **5** | **12** |
| Andrea Montalto |  | 1 |  | 1 |
| Antonio Bozzani | 1 |  |  | 1 |
| Domenico Angiletta | 1 |  |  | 1 |
| Emanuele Gatta | 1 |  |  | 1 |
| Fabio Verzini | 1 |  |  | 1 |
| Gian Franco Veraldi | 1 |  |  | 1 |
| Giovanni Pratesi |  |  | 1 | 1 |
| Luca Bertoglio |  |  | 1 | 1 |
| Mario Moro |  |  | 1 | 1 |
| Mauro Gargiulo |  |  | 1 | 1 |
| Michele Antonello |  |  | 1 | 1 |
| Santi Trimarchi |  | 1 |  | 1 |
| Japan | **19** | **10** |  | **29** |
| Arakawa Mamoru | 1 |  |  | 1 |
| Banno Hiroshi | 1 | 1 |  | 2 |
| Dai Yamanouchi | 1 | 1 |  | 2 |
| Hiraoka Arudo | 1 |  |  | 1 |
| Hoshina Katsuyuki | 1 |  |  | 1 |
| Ichihashi Shigeo | 1 | 1 |  | 2 |
| Inoue Tomoya |  | 1 |  | 1 |
| Ishii Hirohito |  | 1 |  | 1 |
| Kawajiri Hidetake | 1 |  |  | 1 |
| Kikuchi Shinsuke | 1 |  |  | 1 |
| Kodama Akio | 1 |  |  | 1 |
| Kotani Shinsuke |  | 1 |  | 1 |
| Mitsuoka Hiroshi | 1 | 1 |  | 2 |
| Miyamoto Shinji | 1 |  |  | 1 |
| Morikage Noriyasu | 1 |  |  | 1 |
| Morisaki Koichi | 1 |  |  | 1 |
| Ohki Takao | 1 | 1 |  | 2 |
| Shimamura Kazuo | 1 | 1 |  | 2 |
| Shimizu Hideyuki | 1 |  |  | 1 |
| Washiyama Naoki | 1 | 1 |  | 2 |
| Yamanaka Katsuhiro | 1 |  |  | 1 |
| Yamaoka Terutoshi | 1 |  |  | 1 |
| Netherlands | **4** |  | **2** | **6** |
| Foeke Nauta |  |  | 1 | 1 |
| Jan Heyligers | 1 |  |  | 1 |
| Jan-Willem Elshof | 1 |  |  | 1 |
| Kak Khee Yeung | 1 |  |  | 1 |
| Ozan Yazar | 1 |  |  | 1 |
| Vincent Jongkind |  |  | 1 | 1 |
| New Zealand | **2** |  | **1** | **3** |
| Anastasia Dean | 1 |  | 1 | 2 |
| Manar Khashram | 1 |  |  | 1 |
| Saudi Arabia |  | **1** |  | **1** |
| Omer Abdulgadir Abdulrahim |  | 1 |  | 1 |
| Spain | **3** | **1** | **1** | **5** |
| Ana Cristina Marzo Alvarez |  |  | 1 | 1 |
| Begona Soto | 1 |  |  | 1 |
| Elena Iborra Ortega |  | 1 |  | 1 |
| Lucas Mengibar | 1 |  |  | 1 |
| Rosa Lopez Perez | 1 |  |  | 1 |
| Sweden | **1** | **1** | **1** | **3** |
| Carl Wahlgren | 1 |  |  | 1 |
| Kevin Mani |  | 1 |  | 1 |
| Nuno Dias |  |  | 1 | 1 |
| United Kingdom | **4** | **1** | **2** | **7** |
| Colin Bicknell | 1 |  | 1 | 2 |
| Gregory McMahon | 1 |  |  | 1 |
| Leith Williams | 1 |  |  | 1 |
| Marcus Brooks |  | 1 |  | 1 |
| Said Abisi | 1 |  | 1 | 2 |
| United States of America | **41** | **24** |  | **65** |
| Ali AbuRahma | 1 |  |  | 1 |
| Ali Azizzadeh |  | 1 |  | 1 |
| Amit Dwivedi | 1 |  |  | 1 |
| Animesh Rathore | 1 | 1 |  | 2 |
| Anthony Chau | 1 |  |  | 1 |
| Bjoern Suckow | 1 | 1 |  | 2 |
| Brent D. Marsden | 1 |  |  | 1 |
| Brian Sheehan | 1 |  |  | 1 |
| Cecillia Chin |  | 1 |  | 1 |
| Chandler Long | 1 |  |  | 1 |
| Charles S. Briggs | 1 | 1 |  | 2 |
| Cheong J. Lee | 1 |  |  | 1 |
| Courtney Morgan |  | 1 |  | 1 |
| Dennis Gable | 1 | 1 |  | 2 |
| Eanas Yassa | 1 |  |  | 1 |
| Edic Stephanian | 1 | 1 |  | 2 |
| Elliot Stephenson | 1 |  |  | 1 |
| Enjae Jung | 1 |  |  | 1 |
| G. Charles (Chad) Hughes |  | 1 |  | 1 |
| Gautam Agarwal | 1 |  |  | 1 |
| Grace Wang | 1 | 1 |  | 2 |
| Jae-Sung Cho | 1 | 1 |  | 2 |
| Jane He |  | 1 |  | 1 |
| Jill Colglazier | 1 |  |  | 1 |
| Joel Corvera |  | 1 |  | 1 |
| John Henretta | 1 |  |  | 1 |
| Kendal Endicott | 1 |  |  | 1 |
| Kenneth Tran | 1 | 1 |  | 2 |
| Linda M. Harris | 1 |  |  | 1 |
| Marc Schermerhorn | 1 | 1 |  | 2 |
| Mark Farber | 1 | 1 |  | 2 |
| Matthew Goldman | 1 |  |  | 1 |
| Michael Wilderman |  | 1 |  | 1 |
| Micheal Ayad | 1 |  |  | 1 |
| Middleton Chang | 1 |  |  | 1 |
| Mirza Shadman Baig | 1 |  |  | 1 |
| Nathan Droz | 1 |  |  | 1 |
| Nicolas Mouawad | 1 |  |  | 1 |
| Olamide Alabi | 1 |  |  | 1 |
| P. Michael Davis, Jr. | 1 |  |  | 1 |
| Peter Rossi | 1 |  |  | 1 |
| Phillip S. Moore | 1 |  |  | 1 |
| Sagar Gandhi | 1 |  |  | 1 |
| Sara Zettervall |  | 1 |  | 1 |
| Shahab Toursavadkohi | 1 | 1 |  | 2 |
| Sukgu Han | 1 | 1 |  | 2 |
| Todd Vogel | 1 |  |  | 1 |
| Trissa Babrowski | 1 |  |  | 1 |
| Wei Zhou |  | 1 |  | 1 |
| William Jordan |  | 1 |  | 1 |
| William Yoon |  | 1 |  | 1 |
| Zachary C. Schmittling | 1 |  |  | 1 |
| Zain Shahid |  | 1 |  | 1 |
| Grand Total | **92** | **46** | **24** | **162** |

***Displays Active sites as of 27-Jul-26**

# 3. Supplemental Table 3: GREAT Related Publications

1. Allievi et al. Outcomes Following Extended Thoracic Endovascular Aortic Repair for Type B Aortic Dissection from the Global Registry for Endovascular Aortic Treatment. Interdisciplinary CardioVascular and Thoracic Surgery (ICVTS). 2025. 40(7): p.ivaf156. https://doi.org/10.1093/icvts/ivaf156

2. Atkins et al. Raised BMI is associated with fewer Type I endoleaks in patients treated with the Gore Excluder device: data from the Global Registry for Endovascular Aortic Treatment (GREAT). Journal of Cardiovascular Surgery. 2023. 64(5):513-520. https://doi.org/10.23736/S0021-9509.23.12572-9

3. Bachoo et al. Early outcome of endovascular aneurysm repair in challenging aortic neck morphology based on experience from the GREAT C3 registry. Journal of Cardiovascular Surgery (JCVS). 2013. 54(5):573-80.

4. Barber et al. IBE Outcomes from GREAT through 5-years. Journal Vascular Surgery (JVS). 2026. . https://doi.org/10.1016/j.jvs.2026.06.062

5. Barry et al. Excluder Stent Graft-Related Outcomes in Patients with Aortic Neck Anatomy Outside of Instructions For Use (IFU) within the Global Registry for Endovascular Aortic Treatment (GREAT): Mid-term Follow-Up Results. Annals of Vascular Surgery (Ann Vasc Surg). 2021. 76:222-231. https://doi.org/10.1016/j.avsg.2021.04.032

6. Baxter et al. Outcomes of Open Versus Percutaneous Access for Patients Enrolled in the GREAT Registry. Annals of Vascular Surgery (Ann Vasc Surg). 2021. 70:370-377. https://doi.org/10.1016/j.avsg.2020.06.033

7. Bischoff et al. Long-Term Real-World Performance of the GORE® EXCLUDER® AAA Endoprosthesis featuring C3® Delivery System: 10-Year Results from the European C3 Module of the GREAT Registry. European Journal of Vascular and Endovascular Surgery (EJVES). 2025. . https://doi.org/10.1016/j.ejvs.2025.11.044

8. Bissacco et al. Differences in Mid-Term Outcomes Between Patients Undergoing Thoracic Endovascular Aortic Repair for Aneurysm or Acute Aortic Syndromes: Report From the Global Registry for Endovascular Aortic Treatment. Journal of Endovascular Therapy. 2022. 29(5):731-738. https://doi.org/10.1177/15266028211064819

9. Boutrous et al. Predictors of Aneurysm Sac Shrinkage Utilizing a Global Registry. Annals of Vascular Surgery (Ann Vasc Surg). 2021. 71:40-47. https://doi.org/10.1016/j.avsg.2020.08.110

10. Briggs et al. Anatomic and clinical characterization of the narrow distal aorta and implications after endovascular aneurysm repair. Journal Vascular Surgery (JVS). 2018. 68(4):1030-1038.e1. https://doi.org/10.1016/j.jvs.2017.12.073

11. Charlton-Ouw et al. Aortic endograft sizing and endoleak, reintervention, and mortality following endovascular aneurysm repair. Journal Vascular Surgery (JVS). 2021. 74(5):1519-1526.e2. https://doi.org/10.1016/j.jvs.2021.04.045

12. D'oria et al. Incidence, predictors, and prognostic impact of in-hospital serious adverse events in patients ≥75 years of age undergoing elective endovascular aneurysm repair. Surgery. 2023. 173(4):1093-1101. https://doi.org/10.1016/j.surg.2022.11.006

13. Delaney et al. The Impact of Degenerative Connective Tissue Disorders on Outcomes Following Endovascular Aortic Intervention in the Global Registry for Endovascular Aortic Treatment. Annals of Vascular Surgery (Ann Vasc Surg). 2022. 79:81-90. https://doi.org/10.1016/j.avsg.2021.07.013

14. Gable et al. Endovascular treatment for thoracic aortic disease from the Global Registry for Endovascular Aortic Treatment. Journal Vascular Surgery (JVS). 2024. 79(5):1044-56. https://doi.org/10.1016/j.jvs.2023.12.040

15. Giese et al. Five Year Outcomes for Bell Bottom, Iliac Branch Endoprosthesis, and Coil and Cover Approaches from the GREAT Registry. Journal Vascular Surgery (JVS). 2024. S0741-5214(24)00272-6. https://doi.org/10.1016/j.jvs.2024.01.212

16. Grassi et al. Endovascular repair of descending thoracic aortic aneurysms-a mid-term report from the Global Registry for Endovascular Aortic Treatment (GREAT). European Journal of Cardio-Thoracic Surgery (EJCTS). 2022. 61(2):357-364. https://doi.org/10.1093/ejcts/ezab366

17. Howard et al. Editor's Choice - Influence of Proximal Aortic Neck Diameter on Durability of Aneurysm Sealing and Overall Survival in Patients Undergoing Endovascular Aneurysm Repair. Real World Data from the Gore Global Registry for Endovascular Aortic Treatment (GREAT). European Journal of Vascular and Endovascular Surgery (EJVES). 2018. 56(2):189-199. https://doi.org/10.1016/j.ejvs.2018.03.027

18. Kainth et al. Outcomes after endovascular reintervention for aortic interventions. Journal Vascular Surgery (JVS). 2022. 75(3):877-883.e2. https://doi.org/10.1016/j.jvs.2021.08.090

19. Loa et al. The Gore Global Registry for Endovascular Aortic Treatment: Objectives and Design. Annals of Vascular Surgery (Ann Vasc Surg). 2016. 31:70-6. https://doi.org/10.1016/j.avsg.2015.08.024

20. Lomazzi et al. Five-year sex-related outcomes of thoracic endovascular aortic repair in the Global Registry for Endovascular Aortic Treatment. Journal Vascular Surgery (JVS). 2023. 78(3):604-613.e4. https://doi.org/10.1016/j.jvs.2023.05.027

21. Lomazzi et al. Gender Related Access Complications After TEVAR: Analysis from the Retrospective Multicentre Cohort GORE® GREAT Registry Study. European Journal of Vascular and Endovascular Surgery (EJVES). 2020. 60(2):203-209. https://doi.org/10.1016/j.ejvs.2020.04.015

22. Long et al. Five-Year Results for Abdominal Aortic Aneurysm Repair with the GORE® EXCLUDER® Device: Insights from the Gore Global Registry for Endovascular Aortic Treatment (GREAT). Annals of Vascular Surgery (Ann Vasc Surg). 2024. 0/0/0. https://doi.org/10.1016/j.avsg.2024.03.016

23. Marcos et al. Five Year Post-Endovascular Aneurysm Repair Aneurysm Sac Evolution in the GREAT Registry: an Insight in Diabetics Using Propensity Matched Controls. European Journal of Vascular and Endovascular Surgery (EJVES). 2023. S1078-5884(23)00878-X. https://doi.org/10.1016/j.ejvs.2023.10.033

24. Meisenbacher et al. Influence of Adherence to the Instructions for Use on the Outcomes of endovascular treatment of thoracic aortic aneurysms and penetrating aortic ulcers - an analysis from the Gore Global Registry for Endovascular Aortic Treatment (GREAT). Annals of Vascular Surgery (Ann Vasc Surg). 2026. 128: 524-536. https://doi.org/10.1016/j.avsg.2026.02.056

25. Milner et al. Apples and Oranges? Methodological Caveats in Comparing the GREAT and ENGAGE study Outcomes. European Journal of Vascular and Endovascular Surgery (EJVES). 2025. . https://doi.org/10.1016/j.ejvs.2025.09.070

26. Mwipatayi et al. Long-term impact of dyslipidemia on sac dynamics after endovascular aortic repair for abdominal aortic aneurysms in the GREAT registry: An insight in dyslipidemia using propensity matched controls. Vascular. 2025. 0/0/0. https://doi.org/10.1177/17085381251377316

27. Mwipatayi et al. Sex-Related Outcomes After Endovascular Aneurysm Repair Within the Global Registry for Endovascular Aortic Treatment. Annals of Vascular Surgery (Ann Vasc Surg). 2020. 67:242-253.e4. https://doi.org/10.1016/j.avsg.2020.02.014

28. Payne et al. Five-Year Outcomes of Endovascular Treatment for Aortic Dissection From the Global Registry for Endovascular Aortic Treatment. Journal Vascular Surgery (JVS). 2024. S0741-5214(24)01234-5. https://doi.org/10.1016/j.jvs.2024.05.055

29. Pedersen et al. Long-term outcomes of endovascular aneurysm repair in standard versus narrow aortic bifurcation. Journal Vascular Surgery (JVS). 2025. 83(2): 422-429. https://doi.org/10.1016/j.jvs.2025.08.025

30. Piazza et al. Early and Long-Term Outcomes of Endovascular Aortic Repair in Young and Low Surgical Risk Patients in the Global Registry for Endovascular Aortic Treatment. Journal of Endovascular Therapy. 2022. 29(2):248-257.. https://doi.org/10.1177/15266028211045703

31. Piazza et al. Incidence and Predictors of Neurological Complications Following Thoracic Endovascular Aneurysm Repair in the Global Registry for Endovascular Aortic Treatment. European Journal of Vascular and Endovascular Surgery (EJVES). 2019. 58(4):512-519. https://doi.org/10.1016/j.ejvs.2019.05.011

32. Piffaretti et al. Descending thoracic aortic diameter is a predictor of poor outcome after endovascular aortic repair.. European Journal of Cardio-Thoracic Surgery (EJCTS). 2026. ezag108. https://doi.org/10.1093/ejcts/ezag108

33. Piffaretti et al. Endovascular Treatment of Infected Aortic Aneurysms: A Retrospective Multicenter Analysis from the GORE® GREAT Registry Study. International Journal of Endovascular Treatment and Innovative Techniques. 2024. 5(2):49-56. https://doi.org/10.61797/ijetit.v5i2.347

34. Piffaretti et al. Thoracic endovascular stent graft repair for ascending aortic diseases. Journal Vascular Surgery (JVS). 2019. 70(5):1384-1389.1. https://doi.org/10.1016/j.jvs.2019.01.075

35. Potter et al. Early Thoracic Endovascular Aortic Repair for Acute Type B Dissection Is Associated With Increased Complications: Results From the GREAT Registry. Annals of Vascular Surgery (Ann Vasc Surg). 2015. 118:1-10. https://doi.org/10.1016/j.avsg.2025.03.020

36. Ribieras et al. Race-based outcomes of thoracic aortic aneurysms and dissections in the Global Registry for Endovascular Aortic Treatment. Journal Vascular Surgery (JVS). 2023. 78(5):1190-1197.e2. https://doi.org/10.1016/j.jvs.2023.07.004

37. Sayed et al. Mid-term outcomes of endovascular aneurysm repair in challenging aortic neck anatomy based on experience from the GREAT C3 registry. Journal of Cardiovascular Surgery (JCVS). 2020. 61(5):610-616. https://doi.org/10.23736/S0021-9509.19.10857-9

38. Schneider et al. Outcomes of the GORE Iliac Branch Endoprosthesis in clinical trial and real-world registry settings. Journal Vascular Surgery (JVS). 2019. 69(2):367-377.e1. https://doi.org/10.1016/j.jvs.2018.05.200

39. Shiraev et al. Sizing Considerations for GORE Excluder in Angulated Aortic Aneurysm Necks. Annals of Vascular Surgery (Ann Vasc Surg). 2018. 49:152-157. https://doi.org/10.1016/j.avsg.2017.11.051

40. Shutze et al. The incidence and effect of noncylindrical neck morphology on outcomes after endovascular aortic aneurysm repair in the Global Registry for Endovascular Aortic Treatment. Journal Vascular Surgery (JVS). 2018. 68(6):1714-1724. https://doi.org/10.1016/j.jvs.2018.03.394

41. Spinelli et al. Endovascular treatment of complicated versus uncomplicated acute type B aortic dissection. Journal of Thoracic and Cardiovascular Surgery (JTCS). 2023. 165(1):4-13.e1. https://doi.org/10.1016/j.jtcvs.2021.01.027

42. Tjaden et al. Outcomes from the Gore Global Registry for Endovascular Aortic Treatment in patients undergoing thoracic endovascular aortic repair for type B dissection. Journal Vascular Surgery (JVS). 2018. 68(5):1314-1323. https://doi.org/10.1016/j.jvs.2018.03.391

43. Verhoeven et al. Real-world performance of the new C3 Gore Excluder stent-graft: 1-year results from the European C3 module of the Global Registry for Endovascular Aortic Treatment (GREAT). European Journal of Vascular and Endovascular Surgery (EJVES). 2014. 48(2):131-7. https://doi.org/10.1016/j.ejvs.2014.04.009

44. Walsh et al. Survival after endovascular therapy in patients with ruptured thoracic aortic diseases: Results from the Global Registry for Endovascular Aortic Treatment Registry. Journal Vascular Surgery (JVS). 2020. 72(5):1544-1551. https://doi.org/10.1016/j.jvs.2020.02.022
